# The role of pre-existing left-sided valvular heart disease in the prognosis of patients with acute myocardial infarction

**DOI:** 10.3389/fcvm.2024.1465723

**Published:** 2024-11-19

**Authors:** Tamilla Muzafarova, Zuzana Motovska

**Affiliations:** Cardiocenter, Third Faculty of Medicine, Charles University and University Hospital Vinohrady, Prague, Czechia

**Keywords:** aortic stenosis, mitral regurgitation, myocardial infarction, outcome, prognosis

## Abstract

Acute myocardial infarction (AMI) and valvular heart disease (VHD) are the leading causes of cardiovascular morbidity and mortality. The epidemiology of VHD has changed in recent decades with an aging population, increasing risk factors for cardiovascular disease and migration, all of which have a significant implifications for healthcare systems. Due to common pathophysiological mechanisms and risk factors, AMI and VHD often coexist. These patients have more complicated clinical characteristics, in-hospital course and outcomes, and are less likely to receive guideline-directed therapy. Because of the reciprocal negative pathophysiological influence, these patients need to be referred to VHD specialists and further discussed within the Heart team to assess the need for earlier intervention. Since the results of the number of studies show that one third of the patients are referred to the heart teams either too early or too late, there is a need to better define the communication networks between the treating physicians, including internists, general practitioners, outpatient cardiologists and heart teams, after the discharge of patients with pre-existing VHD and AMI.

## Graphical Abstract

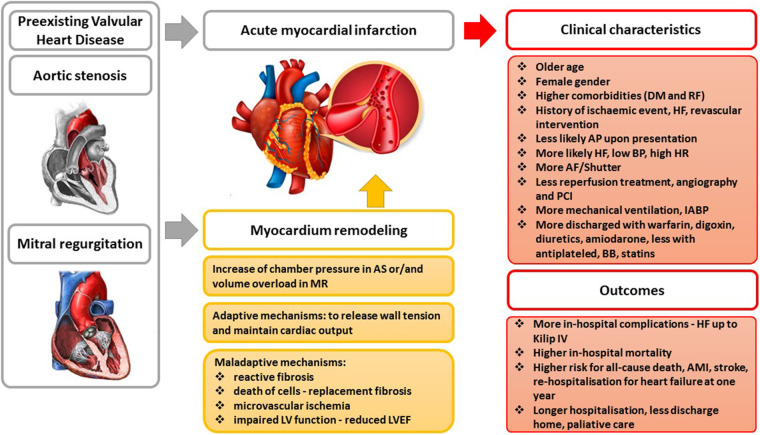

AS, aortic stenosis; MR, mitral regurgitation; AMI, acute myocardial infarction; LVEF, left ventricular ejection fraction; DM, diabetes mellitus; RF, renal failure; HF, heart failure; BP, blood pressure; HR, heart rate; AF, atrial fibrillation; PCI, percutaneous coronary intervention; IABP, intra-aortic balloon pump.

## Introduction

Acute myocardial infarction (AMI) is the most severe manifestation of coronary artery disease (CAD). The increased use of evidence-based therapies has contributed significantly to the reduction in mortality from CAD over the last few decades (1). However, AMI affects more than 7 million people each year across the globe (2). The epidemiology of valvular heart disease (VHD) has changed over the past decade, and the burden of VHD is expected to increase. With population growth, aging and increasing cardiovascular risk factors, VHD is a rising problem and prevalence could double by 2030 (3). In addition, increasing migration flows contribute to the higher incidence of cardiovascular morbidity, including VHD. In the last 15 years, international migration has increased to 272 million, representing 3.5% of the world's population (4). The prevalence of cardiovascular risk factors (hypertension, diabetes, obesity, dyslipidemia) is higher in migrants than in natives (5). Migrants are exposed to a changing environment, lifestyles, behaviors and social changes that may be the risk factors for cardiovascular disease (5). Given the common risk factors and underlying pathophysiological mechanisms, CAD and VHD often coexist (6). VHD often complicate AMI, and much of the recent literature has focused on studying valvular complications of AMI, but there are very few recent data regarding the clinical characteristics and outcomes of patients with acute coronary syndrome (ACS) and pre-existing VHD (7, 8). This coexistence occurs in approximately 5% of patients with AMI and is considered a high-risk cohort, with in-hospital complications and heart failure ranging from mild to cardiogenic shock (7). The availability of new treatments for ACS and the wider use of interventional treatment for VHD have changed the management of these conditions and, consequently, the characteristics of the patients affected. In addition to the high safety and durability of surgical and transcatheter treatment of VHD, survivors are at risk of structural valve damage, thrombosis with the need for reintervention, and cardiac arrest (9). This review aims to provide a contemporary overview of the problem of pre-existing VHD in patients with AMI, the pathophysiological mechanisms of this coexistence, clinical characteristics, management problems, and outcome, which is an important issue due to the increasing burden of VHD.

## Materials and methods

The bibliography for this review was performed using the PubMed search engine up to December 2023, with no restrictions on publication status or start date. A systematic search included all articles that examined outcomes in patients with AMI and a history of VHD, the mechanisms of VHD in AMI, and the coronary circulation. National registries of AMI and ACS patients, nationwide surveys, and studies were evaluated to analyze the prevalence and independent predictive role of pre-existing VHD on AMI outcome. The references cited in the selected articles were also reviewed for additional references. We analyzed left-sided VHD—moderate to severe mitral regurgitation (MR), moderate to severe aortic stenosis (AS), or both. This selection reflects the incidence of VHD in the adult population of developed countries (3), where AMI is one of the leading causes of death (1).

## Results

### The prevalence of VHD in patients with ACS

AS and MR are the most common acquired valvular diseases in developed countries (10–12). The prevalence of AS in patients with AMI ranges from 2.7% up to 16% in octogenarians (13), and pre-exisitng MR is reported from 2.4% up to 13.2% in those >74 years-old (8, 14). The prevalence of degenerative AS is approximately 20% in the >70 years-old Chinese population. Rheumatic AS occurs in 1.86 per 1,000 population in China, 4.54 in Asia, and 1.3 in Bangladesh (15). In developing countries, MR is prevalent in younger adults, due to the rheumatic disease (16). Primary MR caused by mitral valve (MV) prolapse has a prevalence of approximately 2%–4% in both Western and Asian countries. With migration, the prevalence of etiological types of VHD is expected to change, in addition to the increase in cardiovascular risk factors. VHD occurs at approximately the same rate in men and women, but women are less frequently diagnosed in the community, suggesting a diagnostic imbalance leading to a less favorable outcome for women with VHD (3). According to the racial and ethnic studies, the higher prevalence of secondary MR is observed in Black patients compared to White and Asians (17), severe AS is more common in White than Black patients (18).

### Patophysiological mechanisms in aortic stenosis

**Figure 1:**
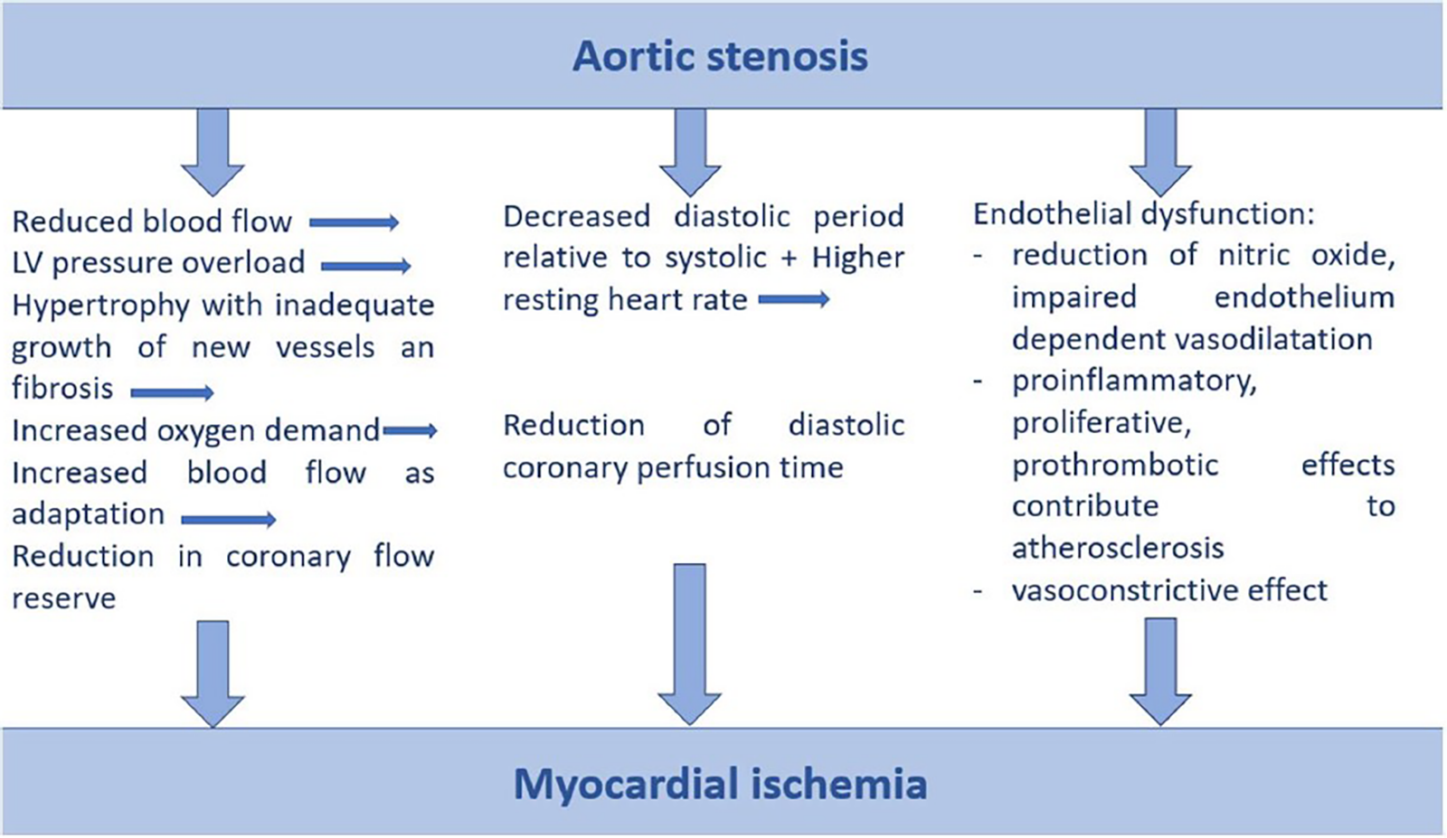
Pathophysiological mechanisms of myocardial ischemia in aortic stenosis. LV, left ventricle.

The leading etiology of AS is degenerative-calcific (81.9%), less commonly rheumatic (11.2%), congenital (5.6%), post-endocarditis (1.3%) (12). The pathophysiology of degenerative AS begins with an initial phase of atherosclerotic changes in the leaflet—thickening, stiffening, progressing to calcification of the leaflets and anulus, impairment of leaflet mobility, leading to valve obstruction (19). As AS progresses from moderate to severe, the reduced aortic valve (AV) area causes a series of structural changes leading to concentric left ventricular hypertrophy in an attempt to increase contractile force and compensate for wall stress in a state of increased afterload (20). Left ventricular hypertrophy results in higher myocardial oxygen demand, however, increased interventricular pressure, which leads to microvascular compression, arteriolar remodeling and fibrosis, limits adequate coronary perfusion, resulting in an oxygen demand/supply mismatch and ischemia (21, 22). An overview of the pathophysiological mechanisms leading to ischemia in patients with AS is shown in Figure 1. The pathogenesis of AS is a complex dynamic process, resulting from the interplay between endothelial system, inflammation, fibrosis and calcification (23). The initial phase develops under the influence of biomechanical factors—oscillatory shear stress causing valvular endothelial dysfunction with diffusion of lipids and infiltration of immune cells causing the local inflammatory response (24–26). The immune cells and oxidized lipids diffuse into the vasculature of AV and promote the release of proangiogenic factors, leading to neoangiogenesis, which may cause intraleaflet haemorrhage (27). Inflammatory cells with myofibroblasts secrete matrix metalloproteinases that promote extracellular matrix remodeling and fibrotic modification of the valve; these myofibroblastic cells are further transformed into osteoblastic cells under the influence of inflammatory cytokines, leading to valve calcification (23). Increasing evidence supports the important role of the hemostatic system (platelets and coagulation system) in the pathogenesis of natural AV stenosis and its progression. The above mentioned pathogenic factors—biomechanical stress, endothelial dysfunction, intravalvular inflammation, neoangiogenesis and osteochondrogenic differentiation—lead to activation of hemostasis with a prothrombotic effect (28).

It is known that AS and CAD often coexist, they share the same pathogenic mechanisms such as lipid deposition, inflammation, osteopontin production, and the same risk factors such as smoking, dyslipidemia, diabetes, and arterial hypertension (29–31), and the progression of CAD and AS is associated with age (32). AMI promotes a series of pathological changes in the AVwith increased collagen production and thickening, leading to its remodeling and accelerating the progression of AS (33). Myocardial ischemia causes fibrosis of the myocardium, leading to coronary microvascular dysfunction (34). Thus, according to the cascade of pathophysiological mechanisms described above, patients with AMI and pre-existing AS are a high-risk group with an unfavourable long-term prognosis.

### Patophysiological mechanisms in mitral regurgitation

Mitral regurgitation can be generally divided into primary and secondary forms. The leading cause of primary MR is degenerative disease of MV, which results from abnormalities of the MV apparatus and is most commonly (in approximately 60%) developed as a result of MVprolapse (fibroelastic deficiency, Barlow's disease with myxomatous leaflets) and less commonly (in approximately 12%) as a result of rheumatic disease and infective endocarditis (3, 35). Degenerative MR is characterized by a variety of morphological changes in MV, including chordal elongation, thinning and rupture, leaflet tissue expansion and annular dilation (36). Damage to one or more components of the MV leads to reverse blood flow and volume overload of the left ventricle. Preload is increased with MR, but afterload remains normal as excess blood volume from the left ventricle returns to the left atrium. These pathophysiological features of loading in primary MR lead to a unique type of remodeling with the highest radius/thickness ratio and lowest mass/volume ratio compared to other types of VHD (37). A larger left ventricular volume allows the forward stroke volume to increase, compensating for the blood volume loss caused by regurgitation. Due to the relatively thin wall of the remodeled left ventricle, diastolic function remains supernormal (38). This compensatory phase allows the release of wall tension and the maintenance of cardiac output. In chronic MR, further adaptive mechanisms go through a transitional phase to decompensation, in which chronic left ventricular wall stress leads to microvascular ischemia, cell death with replacement fibrosis, and adverse myocardial remodeling that impairs left ventricular function, and resulting in dilation, reduced contractility, and reduced left ventricular ejection fraction (LVEF) (39, 40).

Secondary MR develops in a structurally normal or near-normal MV with impaired systolic coaptation between the anterior and posterior leaflets as a result of left ventricular dysfunction or annular dilation due to ischaemic, non-ischaemic cardiomyopathy or atrial dilation (41). Ischemic etiology may cause rupture or displacement of the papillary muscles due to wall motion abnormalities of the underlying segments, or dilation and loss of contractility of the mitral annulus (42). Restriction of leaflet motion leads to incomplete closure of the MV. The pathophysiological mechanisms in secondary ischemic MR are more complex than in structural MR because left ventricular dysfunction precedes MR (43). Compensatory mechanisms after AMI are less effective because the increased MR preload is not accompanied by increased contractility. Chronic volume overload of the left ventricle, which has a lower compliance, leads to higher end-diastolic pressure in of left ventricle and left atrium, followed by their enlargement, pulmonary hypertension and heart failure (44). Enlarged left ventricle causes greater tethering of the mitral leaflets, which promotes the progression of MR.

Other causes of secondary MR include atrial remodeling and isolated annular dilation in the setting of atrial fibrillation and/or heart failure with preserved LVEF (45). Atrial fibrillation and heart failure with preserved LVEF are increasing in prevalence and share common clinical features and pathophysiological mechanisms, with diastolic dysfunction, dysregulated neurohumoral regulation (activation of the renin-angiotensin-aldosterone system and atrial natriuretic peptide) leading to atrial dilation and fibrosis, resulting in isolated annular dilation (46).

The role of varioius pathophysiological mechanisms in MR has been studied. The alterations in hemostasis leading to a prothrombotic state in patients with MR have been discussed since the 1980s, but the results of the studies are controversial, probably due to the wide range of heterogeneous patients with MR (47–49). It has been shown that even mild to moderate MR is associated with higher levels of plasma platelet factors and a higher rate of platelet aggregation with an increased risk of thromboembolic events (50). In addition, platelet activity has been shown to increase with the severity of MR (51). The possible mechanism of platelet activation in MR is hemodynamic disturbance due to turbulent flow in the left atrium, leading to aggregation of activated platelets on the damaged surface of the MV, forming a platelet-fibrin thrombus and leading to thromboembolic events (51). MR presenting itself a prothrombotic state, is an adverse comorbidity of AMI, in which platelets play a key pathogenic role. In addition, AMI triggers a number of remodeling mechanisms that may cause progression of existing MR as described above, leading to a worse outcome in these patients (52–55).

### Clinical characteristics

Patients with pre-existing AS, MR and AMI are characterized by a higher comorbidity index, less typical angina on admission, more Killip 3 and 4 heart failure, lower LVEF, lower systolic blood pressure and higher heart rate. They are characterized by a more complicated in-hospital course with atrial fibrillation, cardiogenic shock and acute multiorgan failure (8, 14). Compared with AMI patients without AS, patients with AS are on average older, female, have more comorbidities, higher rates of non-ST-elevation MI and cardiogenic shock (6). Similar characteristics are observed in AMI patients with pre-existing MR; these patients are older, more likely to be female, have a higher Killip class on admission, angiographically higher rates of multivessel disease, a lower post-procedural thrombolysis in myocardial infarction flow grade in the infarct-related artery, and a lower LVEF (56).

### Management

The only treatment to alter the course of AS is surgical (SAVR) or transcatheter (TAVR) replacement of AV. The decision for SAVR or TAVR intervention is based on multidisciplinary assessment by the Heart team, including multimodality imaging evaluation and assessment of symptom onset (57). According to European guidelines, intervention is indicated for symptomatic severe AS with any LVEF, asymptomatic AS with systolic dysfunction or positive exercise test, TAVR is preferred in higher-risk patients who are unsuitable for surgery, SAVR in asymptomatic patients with severe AS and preserved systolic function (58). The introduction of TAVR has significantly changed the management strategies for AS (59, 60). Large registries in the USA, Canada, France and Germany show increasing availability of the procedure, decreasing mortality in patients with AS after the procedure, a trend to expand the indication to intermediate and low-risk patients, and predict a decreasing trend in SAVR (61–63). However, the other side of the wider use of AV replacement is that the survivors are at a risk of valve thrombosis and endocarditis, requiring reintervention (9).

MR can be challenging to manage due to the heterogeneity of etiopathophysiological aspects and its dynamic nature. In primary MR a surgical repair of MV is recommended in symptomatic severe MR with LVEF >30% or asymptomatic but with LVEF 30%–60% or left ventricle end-systolic dimension ≥40 mm. MV repair is preferred to replacement, when possible. The optimal timing of interventional correction of primary MR improves prognosis (64). Transcatheter intervention is an option for the patients with contraindications to surgery or at high risk, after accurate assessment and based on the decision of the multidisciplinary Heart team. A randomized trial comparing transcatheter and surgical intervention showed that although the transcatheter technique is less effective in reducing MR, it is associated with superior safety and improved prognosis (65).

In secondary MR, which has a multifactorial etiology with left atrial and ventricular dysfunction and remodeling, even the milder severity is associated with a worse outcome compared to primary MR. Due to the complexity of secondary MR, intervention is not the first-line therapy. Management is based on guideline-directed medical management of heart failure under the guidance of a multidisciplinary team, including experts in heart failure and electrophysiology (66). The decision of surgical MV repair or replacement of severe MR is based on the pathoanatomic details assesed by multimodality imaging. Mitral valve repair is preferred in degenerative MR (59). Transcatheter replacement of MV is a rapidly evolving interventional approach for the patients with secondary MR. In the multinational registry analysis by Nickening et al, this transcatheter replacement procedure has shown high success, low complications, and improvement in MR severity and clinical symptoms (67).

Despite the poor outcome of untreated MR, the intervention is performed in a small percentage of MR, even if the valvopathy origin is degenerative (65, 68).

In AMI, acute left ventricular remodeling of the infarcted region leads to the progression of the pre-existing mitral regurgitation. Patients with AMI and pre-existing MR require closer monitoring with fluid balance control, echocardiographic assessment of the impact of the new dysfunction on the severity progression of the pre-existing mitral regurgitation, and therapeutic intervention at the first signs of destabilization. First-line therapy includes intravenous diuretics, vasodilators to reduce the regurgitant flow, and stroke volume augmentation to reduce left ventricular afterload (69).

Patients with pre-existing VHD admitted with AMI are characterized by a lower use of guideline-directed therapies. Although they have worse in-hospital outcomes, they are less likely to undergo coronary angiography and percutaneous coronary intervention than AMI patients without significant VHD (8). As shown in the US study of more than 11 million AMI patients, admissions with AS have higher rates of coronary artery bypass graft surgery and SAVR, but significantly lower use of coronary angiography, percutaneous coronary intervention and mechanical circulatory support (6). During hospitalization, patients with pre-existing AS and MR are more likely to receive warfarin, digoxin, diuretics, intravenous inotropic agents, amiodarone, angiotensin-converting enzyme inhibitors, while treatment with beta-adrenergic blockers, antiplatelet agents and statins is less common (8). An overview of management strategies is provided in Tables 1, 2, but the question of more frequent follow-up and earlier evaluation of intervention in AMI patients with pre-existing VHD remains open for discussion.

**Table 1:** Management of aortic stenosis.

Aortic stenosis		
Follow-up controls: mild 3–5 years, moderate 1–2 years, severe 6–12 months		
Intervention		
Severe symptomatic AS (regardless LVEF)		
Severe asymptomatic AS with reduced LVEF <50% or undergoing cardiac surgery for other indications		
Severe asymptomatic AS and decreased exercise tolerance, serum brain natriuretic peptide >3× normal, or blood flow velocity across the AV that increases by ≥0.3 m/s per year		
Very severe AS with a transvalvular velocity of ≥5 m/s		
The choice between surgical and transcatheter AV intervention is based on clinical, anatomical, and procedural factors, surgical risk, patient frailty, comorbidities and patient preferences		
Transcatheter AV replacement	Surgical AV replacement	Baloon aortic valvotomy
STS-PROM/EuroSCORE II >8%, Age ≥75 years, contraindications for surgery	STS-PROM/EuroSCORE II <4%, Age <75, unfavorable anatomy for transfemoral TAVR	Bridge to AV replacement in hemodynamically unstable patients

AS, aortic stenosis; AV, aortic valve; LVEF, left ventricle ejection fraction; STS-PROM, Society of Thoracic Surgeons 30-day Predicted Risk of Mortality score; EuroSCORE, The European System for Cardiac Operative Risk Evaluation; TAVR, transcatheter aortic valve replacement.

**Table 2:** Management of mitral regurgitation.

Mitral regurgitation				
Follow-up controls: mild 3–5 years, moderate 1–2 years, severe 6–12 months				
Primary mitral regurgitation				
Step 1	Step 2	Step 3	Step 4	
What is the mechanism of MR?	How severe is MR?	Does MR meet criteria for intervention?	Which type of intervention to choose? Surgical repair or MitraClip?	
Leaflet morphology and motion	Anamnesis, physical examination	ESC, ACC/AHA guidelines	Surgical repair is indicated in patients with low operative risk and with symptomatic severe MR, in severe asymptomatic MR with left ventricular dysfunction.	
Subvalvular involvement				
	Quantitative and qualitative imaging parameters			
Annulus dilation, calcification				
			MitraClip is considered in symptomatic severe MR with high operative risk.	
The impact of MR on LV and LA size, function				
Secondary mitral regurgitation				
Step 1	Step 2	Step 3	Step 4	Step 5
What is the mechanism of MR?	How severe is MR?	End stage heart failure?	Does MR meet criteria for intervention?	Which type of intervention to choose? Surgical repair/replacement or MitraClip?
	+ Anamnesis, physical examination			
	+ quantitative and qualitative imaging parameters			
Leaflet morphology and motion	In symptomatic severe MR etiological therapies, the first line - guideline-directed medical therapy / cardiac resynchronization therapy	In persistent symptomatic severe MR with end-stage heart failure - advanced heart failure therapies; without end-stage heart failure - evaluation of intervention according to guidelines	ESC, ACC/AHA guidelines	Ischemic MR:
				In symptomatic severe MR unresponsive to therapy for LV, replacement of MV should be considered as the first line.
Subvalvular involvement			Symptomatic severe MR even after treatment, symptoms are from the MR and not the cardiomyopathy, surgical MV replacement can be considered.	
Annulus dilation				
LV and LA size, function				
Coronary angiography to evaluate the cause of systolic dysfunction or inferior wall motion abnormality.				
				In MR without inferobasal aneurysms and those with a smaller sized LV, repair of MV may be considered
				MitraClip efficacy for high-risk patients is unproven.
				In nonischemic secondary MR, there is no evidence supporting MV intervention for prolongation of life or prevention of further LV dysfunction.

MR, mitral regurgitation; MV, mitral valve; LV, left ventricle; LA, left atrium, ESC, European Society of Cardiology; ACC, American College of Cardiology; AHA, American Heart Association.

### Outcome

Patients with both moderate-to-severe MR, AS and AMI have worse outcomes. In the Elderly-ACS 2 study, AMI patients with AS had a threefold and AMI patients with MR a twofold increased risk of all-cause death, AMI, stroke and rehospitalization for heart failure at one year (14). Pre-existing AS in AMI patients was associated with longer hospital stay, more frequent palliative care, do-not-resuscitate status, higher in-hospital mortality and less frequent discharge to home (6). A number of studies have shown the association between the severity of VHD and outcome. Even the presence of mild MR at baseline is a strong independent predictor of reduced survival after primary percutaneous coronary intervention (56), which may be explained by the impact of myocardial ischemia on MR progression. The other study reported the association between AS severity and outcome observed in normal LVEF, in patients with reduced LVEF the outcome was poor across all AS severities, as there was a higher risks of in-hospital Killip ≧ 3 heart failure, major bleeding, and acute kidney injury (70).

## Conclusion

AMI and VHD are the leading causes of cardiovascular morbidity and mortality. Pre-existing left VHD is often encountered in AMI patients due to the shared pathophysiological mechanisms and risk factors. These patients have more complicated clinical presentation, in-hospital course, and worse outcome, are less likely to receive guideline-directed therapy, and have lower rates of coronary angiography and percutaneous coronary intervention. The high number of patients with AMI, the increasing burden of VHD with an aging population, the growing risk factors for cardiovascular disease and migration have a significant impact on the healthcare system. The evidence of poorer prognosis in patients with this coexistence requires precise management strategies both during hospitalization and at follow-up. VHD should be accurately assesed during the hospitalization of patients with AMI.

Because of the negative impact of AMI on the progression of VHD, it is necessary to more frequently and accurately assess the progression of VHD. The assessment of patient-reported symptoms is important but not sufficient. We need to perform more accurate evaluation using echocardiography and, if necessary, multimodal imaging techniques, including two- and three-dimensional echocardiography, cardiovascular magnetic resonance and computed tomography. The indication for intervention should be discussed within the multidisciplinary Heart team, including clinical and interventional cardiologists, surgeons and imaging specialists. As reported by Iung et al. in the Euro Heart Survey, currently one third of patients with AS are referred for intervention either early or late (12). This suggests the need to better define the communication networks between physicians caring for patients, including internists, outpatient cardiologists and multidisciplinary Heart teams after the discharge of patients with pre-existing VHD and AMI, leading to improved patient care. Primary care physicians should pay more attention to VHD patients after MI. Clearly defined referral standards and protocols will help them to assess the need for timely referral of these patients to cardiologists. In addition, the development of special virtual platforms and applications to link primary care physicians and cardiologists will be of great interest and importance. The VHD-AMI patients should undergo echocardiographic assessment before discharge and early after discharge to define the progression of VHD. Patient involvement and education about the diagnosis is important not only for patient adherence to follow-up, but also for improving communication between primary care physicians and cardiologists.
